# Immune-Based Prevention and Therapy Against Coccidioidomycosis: Current and Emerging Approaches

**DOI:** 10.3390/jof12030214

**Published:** 2026-03-17

**Authors:** Nawal Abdul-Baki, Reimi Navarro, Jieh-Juen Yu, Chiung-Yu Hung

**Affiliations:** Department of Molecular Microbiology and Immunology, South Texas Center for Emerging Infectious Diseases, The University of Texas at San Antonio, San Antonio, TX 78249, USA; nawal.abdul-baki@my.utsa.edu (N.A.-B.); reimi.navarro@my.utsa.edu (R.N.); jiehjuen.yu@utsa.edu (J.-J.Y.)

**Keywords:** *Coccidioides*, Valley Fever, vaccines, adjuvants, mRNA, organoids, immunotherapy

## Abstract

Coccidioidomycosis (CM) is a fungal infection caused by the inhalation of airborne arthroconidia released by *Coccidioides* spp. Endemic areas include the southwestern United States, Mexico, and parts of South America. The estimated CM cases exceed 300,000 per year. Current treatment for CM is limited and primarily relies on antifungals such as azoles and Amphotericin B. Moreover, concerns about drug cytotoxicity and rising of azole-resistance underscore the need for alternative or adjunctive immune-based prevention and therapies. This review presents recent advances in immune CM intervention and discusses the potential application of emerging antifungal immunotherapy to treat invasive CM. For preventive vaccination, we reviewed the recent development of subunit protein vaccines and mRNA-based vaccines. Prospects for formulating vaccines with potent adjuvants and delivery systems to enhance protective immunity against CM are also provided. For immunotherapy, we reviewed recent reports of antifungal treatment with immunomodulators, CAR-cells and checkpoint inhibitors. Finally, we discuss the application of experimental animal and in vitro models for advancing vaccine and immunotherapeutic development for CM.

## 1. Background

Coccidioidomycosis (CM) is an emerging respiratory fungal disease that has increased in prevalence over the last decade. CM is one of the most expensive mycoses, costing upwards of 3.9 billion annually [1]. The etiologic agents of this disease, *Coccidioides posadasii* (*C. posadassi*) and *Coccidioides immitis* (*C. immitis*), are endemic to the southwestern United States, certain areas of Mexico and South America, but have been spreading across geographical regions in the past decade [2,3]. When soil is disturbed, arthroconidia are aerosolized and inhaled into the lungs, where they begin isotropic growth into parasitic spherules. Spherules then rupture to release hundreds of endospores that can disperse into distal tissues to continue the parasitic cycle. *Coccidioides* spp. are capable of causing disease in both immunocompetent and immunodeficient individuals. Around 60% of cases are asymptomatic, while 40% present flu-like symptoms, ranging from moderate to severe [4,5]. Of the 40% of patients who present with symptomatic disease, roughly 5–10% develop severe complications or disseminated CM [6].

Risk factors for developing severe disease include underlying immunocompromised conditions (organ transplant, diabetes, and HIV/AIDS), pregnancy, ethnicity, and genetic predispositions. Immunocompromised individuals have a higher propensity to develop disseminated disease [7]. Pregnancy also increases the risk of *Coccidioides* dissemination particularly when infection is acquired during the later stages of gestation [8]. Certain ethnicities are more susceptible, such as Hispanics, African Americans and Pacific Islanders, as compared to Caucasians [9]. Other human genetic determinants associated with CM severity were recently reviewed by Galgiani et al. [10]. However, the most defined host innate and adaptive immunity against CM was demonstrated in the murine CM model [9,11,12].

**Figure 1:**
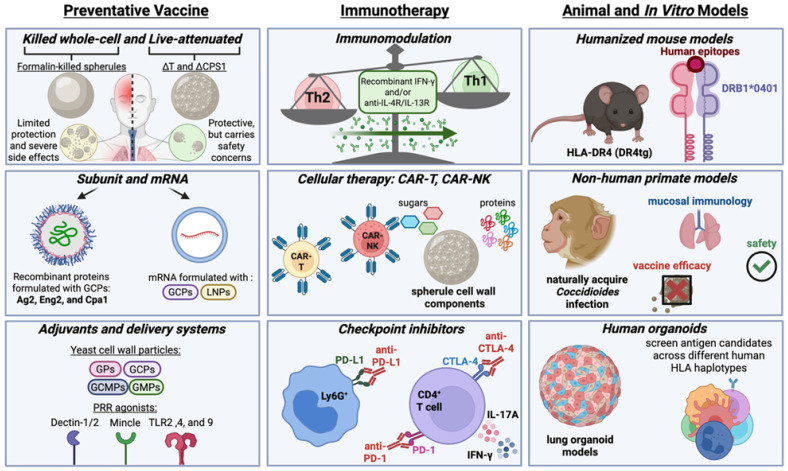
Considerations for *Coccidioides* Vaccine and Therapeutic Development. The left panel demonstrates killed whole-cell and live attenuated vaccines, subunit and mRNA vaccines, and adjuvant delivery systems. The middle panel explores new and upcoming immunotherapies including immunomodulation, cellular therapy, and checkpoint inhibition. The right panel shows humanized mouse models, non-human primate models, and human organoid models as tools for vaccine and therapeutic development. GCPs, Glucan-Chitin-Particles; LNPs, Lipid Nanoparticles; GPs, Glucan-Particles; GCMPs, Glucan-Chitin-Mannan-Particles; GMPs, Glucan-Mannan-Particles; PRR, Pattern Recognition Receptor.

Interventions for fungal diseases typically involve preventive vaccination and therapeutic antifungal medications. It is well documented that recovered CM patients develop lifelong immunity, suggesting that vaccination against this fungal disease is feasible. Despite efforts since the 1960s to develop an efficacious and safe vaccine for Valley Fever, no clinical vaccine has yet reached the market. For therapy, current clinical CM treatment options are limited to azoles such as fluconazole and itraconazole, and the polyene amphotericin B [13,14,15]. Efforts to identify new anti-*Coccidioides* drugs often involve high-throughput screening of repurposing libraries [16,17,18]. However, due to cytotoxicity and the rise of azole-resistance to current antifungal medicine as well as slow progress in new drug development, immunotherapy has emerged as a potential alternative/adjunctive therapy for managing CM. In this review, we highlight preclinical advances in (1) preventive vaccine development, antigen discovery, and adjuvant delivery technology; (2) emerging immunotherapies including immunomodulators, checkpoint inhibitors, and cell-based therapy; and (3) application of experimental models for advancing vaccine and immunotherapeutic development (Figure 1).

## 2. Preventive Vaccine

Rational vaccine development prioritizes several areas, as listed by the National Institute of Allergy and Infectious Diseases (NIAID), such as addressing gaps in basic research, developing tools to support vaccine research, and strategizing to advance vaccine design. Although no licensed vaccine approved for CM, results from past and current collaborative vaccine research in the field have provided insightful values of various adaptable vaccine platforms and shed light on protective immunity critical for further human CM vaccine development.

### 2.1. Killed Whole-Cell and Live-Attenuated Vaccines

Most effective vaccines against microbial pathogens have been developed using killed whole cells or live-attenuated strains. In the 1960s, a killed whole-cell vaccine generated using formalin-killed spherules (FKS) was found to be efficacious in preventing lethal *Coccidioides* infections in mice and cynomolgus monkeys, which led to its evaluation in a phase III human trial [19,20,21]. However, clinical development was halted after the trial due to concerns about limited protective efficacy and adverse side effects, such as fever, inflammation, and pain at the injection sites [21].

Subsequent cellular vaccine development has focused on attenuated live vaccines. The most advanced *Coccidioides* vaccine candidates, ∆cts2/∆ard1/∆cts3 (∆T) and ∆CPS1, are derived from attenuated strains lacking genes essential for endosporulation [22,23]. The protective capacity of the ∆T strain was evaluated in BALB/c and C57BL/6 mice challenged with the C735 *Cp* clinical isolate; it was found to reduce the fungal burden in the lung by roughly 5-fold and 7-fold, respectively, with prolonged survival [22]. Moreover, histopathological assessment revealed that mice administered with the ∆T vaccine had well-defined granulomas with minimal influx of inflammatory cells and spherule initials [22]. Protective pulmonary immunity to *Coccidioides* infection in the ∆T vaccinated mice was associated with early activation of the Th1, Th2, and Th17 pathways [24]. In the same study, Hung et al. further demonstrated that Th17 immunity is critical for vaccine protection, showing that the loss of functional Th17 cells resulted in increased susceptibility to infection in immunized mice [24]. Despite the promise this vaccine held, it was not investigated further as a potential human vaccine due to concerns about the risk of fungal pathogenicity in an immunocompromised population. Despite this drawback, the ∆T strain has been made commercially available to the *Coccidioides* research community as a BSL/2 agent for novel antigen identification, new vaccine development as a comparative vaccine (e.g., immune responses, efficacy), and new anti-CM drug screening as target cells [16,17].

More recently, the ∆CPS1 vaccine was shown to invoke protection in immunocompetent (C57BL/6 and BALB/c) and several primary immunodeficient (STAT4, STAT3, IFNgr1, and Dectin-1 knockout) mouse models [23,25]. These murine protection data suggest that the ΔCPS1 vaccine could benefit humans with immunogenetic risks of disseminated CM. Additionally, the ∆CPS1 vaccine has also been evaluated for potential use in dogs, as they are one of the animals heavily impacted by CM [26]. Shubitz et al. found that dogs administered with two doses of the ∆CPS1 vaccine were protected from developing severe CM [27]. Based on these data, the ∆CPS1 vaccine has garnered interest and funding to advance toward a Phase I clinical trial [28]. This would make it the second vaccine candidate to enter clinical evaluation after the FKS vaccine. Given that recent trends in CM vaccine development have emphasized subunit platforms due to their favorable safety profiles, the ∆CPS1 vaccine will need to address several key limitations. These include demonstrating that the attenuated strain causes minimal reactogenicity in immunocompetent and immunocompromised populations, developing assays to confirm that each production lot is genetically stable over time and does not revert to the pathogenic form, and establishing storage conditions that maintain viability and shelf life.

### 2.2. Subunit Vaccines

Subunit fungal vaccines are a safer and more advantageous alternative to cellular vaccines, as they can be produced through recombinant protein expression or peptide synthesis without the risk of fungal pathogenicity. Their potential has been widely studied not only in *Coccidioides* but also in other pathogenic fungi (*Candida*, *Aspergillus*, *Pneumocystis*, *Blastomyces*, *Histoplasma*, *Cryptococcus)*, demonstrating the promise of this platform [29,30,31,32]. Ideal anti-CM vaccine candidates are proteins or peptides that (1) lack human homologs, (2) are highly expressed during the parasitic phase, (3) contain epitopes that bind to MHC-II molecules, and (4) are preferably present on the fungal cell wall surface. Subsequent formulation of protein/peptide antigens with proper adjuvants (detailed review in Section 2.4) to elicit robust protective cell-mediated Th1 and Th17 immunity is critical for an efficacious CM vaccine [24,33].

Reported preclinical CM subunit vaccines include Antigen-2 [34], Eng2 [31], and rCpa1 [29,35], all formulated with a glucan-chitin-particle (GCP) adjuvant derived from a non-pathogenic yeast, *Rhodotorula mucilaginosa* [36]. Immunization with bacterium- and maize-expressed Antigen 2 (Ag2) protected against pulmonary coccidioidomycosis by reducing 92% and 82% fungal lung burden, respectively [34]. Vaccination of mice with endoglucanase 2 (Eng2), a shared immunodominant antigen among medically important dimorphic fungi, reduced coccidioidal burden and improved the survival rate [31]. More importantly, PBMCs from 16 out of the 20 patients (80%) who recovered from confirmed *C*. *posadasii* infection produced IFN-γ or IL-17 in response to stimulation with the Eng2 protein. These human data suggest that Eng2 is a potent immunogen and that vaccination with Eng2 in humans could potentially generate Eng2-specific memory T cells required for protection against CM.

Unlike the single protein Ag2 and Eng2 subunit vaccines, rCpa1 is a multivalent antigen vaccine consisting of Ag2/Pra, Cs-Ag, Pmp1, and five predicted human epitope peptides derived from Pep1, Amn1, and Plb [29]. Broad-spectrum protection against virulent *C. posadasii* and *C. immitis* strains by the GCP-rCpa1 vaccine has been demonstrated in both conventional C57BL6 and humanized HLA-DR4 (DRB1*0401) transgenic mice [35]. As expected, GCP-rCpa1 protection is associated with the activation of both Th1 and Th17 immunity [29,35]. With the advancement in genomic, proteomic, and T cell epitope prediction tools, identification of new coccidioidal antigens can be greatly accelerated [37,38,39,40,41]. Validation of the immunogenicity of identified vaccine candidates by IFN-γ recall assay using patient PBMCs can provide valuable insights into human relevance for CM vaccine design.

### 2.3. mRNA Vaccines

The COVID-19 pandemic has accelerated the development of mRNA vaccines against infectious diseases [42,43,44]. Recent advancements in mRNA vaccines have led to more robust immune responses, prolonged shelf-life, and reduced production timelines. These improvements highlight their potential for developing effective mRNA vaccines against *Coccidioides* and other fungal pathogens. Indeed, Li et al. reported the development of fungal mRNA vaccines, demonstrating protective efficacy in mice against cryptococcosis [45]. The vaccine was constructed by packaging mRNA encoding *C. neoformans* chitin deacetylase 1 (*CDA1*) in lipid nanoparticles and subsequently coupled with purified fungal capsule as an adjuvant to enhance efficacy. Additionally, Yaniv-Rosenfeld’s group have utilized bioinformatics tools, including T and B cell epitope prediction, simulations that predict immunogenicity and ability to bind to PRRs, and physiochemical properties to design mRNA vaccines towards *Cryptococcus neoformans* and *Trichophyton rubrum* [46,47]. Although promising, follow-up studies need to be conducted to validate their in silico findings. The development of CM mRNA vaccines is a current effort in our lab. The mRNA encoding *Coccidioides* antigens are commercially synthesized. The coding sequences of coccidioidal antigens with an N-terminus His-tag are flanked with the 5′ and 3′ UTR of a human gene and cloned into selected plasmids. These plasmids contain a T7 promoter for in vitro transcription (IVT). The 5′ cap and 3′ poly A tail (>80 bp) are added during the IVT reaction. N1-methyl-pseudouridine, a modified UTP, is incorporated into mRNA using a T7 RNA polymerase during IVT to reduce the immunogenicity of the resulting mRNA. Additionally, a dendritic cell targeting sequence (DCpep) that encodes a 12-mer peptide (FYPSYHSTPQRP) [48] can be included at the C-terminus of the mRNA vaccine construct to enhance antigen presentation. The mRNA construct is then packaged in modified GCP particles which contain a cationic core that can efficiently bind anionic nucleic acid payloads through electrostatic interactions. Subcutaneous delivery of the GCP-mRNA vaccine resulted in the expression of coccidioidal antigens in macrophages and dendritic cells at the injection site and the generation of antigen-specific Th1 and Th17 immunity. Moderate protection was observed, and refinement of the mRNA construct with an alternative packaging/delivery system is currently being explored to further improve protective efficacy against CM.

### 2.4. Adjuvant and Delivery Systems

Adjuvants are immunological agents incorporated into vaccine formulations to enhance and shape antigen-specific immune responses. For regulatory purposes, adjuvants are approved only as part of a complete vaccine, not as standalone agents. To date, the only adjuvants included in FDA-approved vaccines are aluminum salts, AS01_B_, AS03, AS04, CpG 1018, Matrix M, and MF59. These adjuvants are primarily used in antiviral vaccines to promote antiviral immunity by enhancing antibody (Th2), Th1, or CD8^+^ T cell responses. In contrast, for CM, it is well accepted that a strong cell-mediated response consisting of mixed Th1 and Th17 immunity is necessary for protection [24,49,50]. While very few Th17-skewing adjuvants have been reported, their development has become a priority as more diseases highlight the importance of Th17 cells.

To mimic fungal infection-induced host defense, several yeast cell wall particles derived from non-pathogenic fungi, including *Saccharomyces cerevisiae* and *R. mucilaginosa*, have been developed as adjuvants. These particles inherit the ability to stimulate the immune system with fungal PAMPs, including β-glucans, mannan, and chitin [36,51]. Many vaccines formulated with these particles have been shown to effectively amplify Th1/Th17 immunity in animal models of *Coccidioides*, *Cryptococcus*, *Blastomyces*, and *Histoplasma* [29,52,53,54]. As described above in Section 2.2, Glucan-Chitin-particles derived from *R. mucilaginosa* have been used as an adjuvant and a delivery system to activate Eng2- and rCpa1-spectific Th1/Th17 protective immunity against CM [29,31,35]. Although these fungal cell wall adjuvants are effective, their compositional complexity and concerns about batch-to-batch inconsistency may hinder their approval for formulating clinical vaccines.

Other choices for CM vaccine adjuvants include ligands for Macrophage-Inducible C-Type Lectin (Mincle), which have been shown to skew T-helper differentiation towards a Th1 and/or Th17 phenotype [55,56,57]. Chemically defined Mincle agonists have been developed as vaccine adjuvants. Several infectious disease and cancer vaccines containing these adjuvants have been evaluated in clinical trials [58]. For instance, the human response to a chlamydia vaccine, CTH522/CAF01, generated robust Th1 and moderate Th17 immunity [59]. CAF01 is a fully synthetic liposome-based adjuvant system comprising a cationic quaternary ammonium salt and the Mincle ligand trehalose dibehenate (TDB). Mincle ligands can also be co-delivered with other PAMPs to boost the desired adjuvant effect. For example, the cationic adjuvant formulation CAF10b, which activates Mincle and TLR9 via monomycolyl glycerol and CpG2006, respectively, enhanced cell-mediated immunity in mice and non-human primates when combined with the *Mycobacterium tuberculosis* H107e vaccine antigen [60]. Immunization of non-human primates with H107/CAF10b led to robust and sustained IFN-γ and IL-17A responses superior to those induced by CAF01 [60]. H107e/CAF10b is now being evaluated in a phase I clinical trial (NCT06050356) as a tuberculosis vaccine.

A recent review by Moses et al. on fungal vaccines in preclinical studies and clinical trials highlighted additional potential candidate adjuvants for CM subunit vaccine development [61]. For example, aluminum hydroxide which is being evaluated in clinical *Candida* vaccine trials can stimulate human Th1/Th2/Th17 immunity [62]. Aluminum Phosphate (AlPO4), when used as an adjuvant for the *Candida* Als3-Hyr1 fusion protein, stimulated robust Th1/Th17 immune responses in vaccinated mice [63]. Furthermore, with federal funding for research in adjuvant development for vaccines against cancers and infectious diseases, many Th1, Th17 and Th1/Th17 adjuvants have been characterized and listed in The Vaccine Adjuvant Compendium (VAC; established by National Institute of Allergy and Infectious Diseases) searchable database (https://vac.niaid.nih.gov/, accessed on 10 December 2025).

### 2.5. Challenges

While the pre-clinical development of vaccines against CM shows tremendous promise, transitioning these technologies from the bench to the clinic faces substantial economic and logistical limitations. Subunit and live-attenuated vaccines can generally leverage existing manufacturing infrastructure, but modern platforms pose distinct challenges. For example, mRNA vaccines typically require strict ultra-cold chain storage and transportation to ensure lipid nanoparticle stability and RNA integrity. Because CM is highly endemic to arid regions of the southwestern United States, Mexico, and South America, maintaining such a rigorous cold chain presents a substantial logistical hurdle. The economics of vaccine development also create significant obstacles. Since CM is geographically restricted, development of a CM vaccine may be perceived as having limited market potential, which could reduce pharmaceutical investment and slow vaccine development and deployment. However, the endemic range of *Coccidioides* is projected to expand substantially, potentially reaching up to half of the U.S. and resulting in roughly 50% more annual cases by 2095 [64]. Therefore, as the at-risk population grows, the public health need and potential market for a vaccine will likely increase. Additionally, strategies that broaden the vaccine’s applicability, such as development of a pan-fungal vaccine targeting dimorphic fungal pathogens (e.g., *Coccidioides*, *Histoplasma*, and *Blastomyces*) could further incentivize potential investors. In recent decades, vaccine hesitancy has been attributed to reducing vaccine coverage [65]. This coupled with limited public awareness of *Coccidioides* may present challenges for vaccine rollout. Therefore, collaborative efforts with public health agencies to improve education and awareness of CM will be important to support future vaccine implementation.

## 3. Immunotherapy

The limited availability of antifungal drugs and the emergence of antifungal resistance have created more challenges in treating invasive fungal infections. Thus, the development of alternative or adjunctive therapeutic strategies has become an urgent need. With advances in understanding the coordinated immune responses to clear *Coccidioides* infection, immune-based therapies could fill the gaps in managing CM.

### 3.1. Immunomodulation

Protective immunity against CM is strongly linked to Th1 and Th17 CD4^+^ T cell responses in murine models [9,24]. In humans, Th1 immunity is widely recognized as protective, while Th2-skewed responses are associated with debilitating disease progression and prognosis [66]. IFN-γ has been shown to activate human PBMCs to inhibit the development of endospores into spherules [67]. Downregulation of the expression of Th1-associated cytokines (e.g., IFN-γ) by Th2 effector cytokines (e.g., IL-4) may result in poor prognosis in CM. Indeed, Type-2 skewing of CD4^+^ T cells is present in a significant fraction (~20%) of patients with disseminated CM [68] and genetic defects in the IL-12-IFN-γ signaling have been implicated in patients with severe CM [7,68]. These data underscore the importance of mechanistic rationale to personalize patient care. Notably, recombinant IFN-γ therapy has been shown to redirect Th2-dominated immunity in disseminated CM patients toward a Th1 profile, leading to improved clinical responses [69,70]. Alternatively, blocking Th2 polarization with dupilumab, an anti-IL-4R/IL-13R monoclonal antibody, can suppress excessive Th2 responses and restore Th1 immunity [68,71]. When used together, these two approaches were demonstrated to augment protective Th1 responses that lead to the resolution of disease in a patient with severe, life-threatening disseminated CM [66].

Modulation of myeloid immune cells by Colony-Stimulating Factors (CSFs) has been shown to improve antifungal activity. For example, combination therapy with recombinant human M-CSF (Macrophage-CSF) and conventional antifungals resulted in better survival for bone marrow transplantation patients with invasive fungal diseases compared to historical controls (27% vs. 5%) [72]. GM-CSF (Granulocyte-Macrophage-CSF) was also found to enhance the anti-fungal activity of neutrophils and monocytes against various fungal pathogens, including *Candida*, *Aspergillus*, and *Cryptococcus* [73,74]. Up-regulation of GM-CSF has been demonstrated during early immune responses to *Coccidioides* infection [75]; however, its application for CM treatment remains to be evaluated.

### 3.2. Cellular Therapy

Advances in cancer immunotherapy, particularly chimeric antigen receptor (CAR)-T cell technology, have opened opportunities for treating infectious diseases. Fungal infections, which depend heavily on T cell immunity, are prime targets for this cell-targeted therapy.

Studies on invasive fungal pathogens, such as *Aspergillus* and *Cryptococcus*, have demonstrated the feasibility and potential efficacy of CAR-T cell approaches. For *Aspergillus*, CAR-T cells engineered to recognize conserved cell wall proteins or carbohydrates on hyphae and germlings, respectively, were found to exert direct antifungal activity and stimulate cytokine production, thereby limiting hyphal growth and reducing disease severity [76,77]. For cryptococcosis, da Silva et al. developed CD8^+^ CAR-T cells targeting the glucuronoxylomannan (GXM) polysaccharide to improve immune recognition normally hindered by the capsule [78]. These GXM-specific CAR-T cells effectively bind the fungus and secrete key immune mediators such as granzyme and IFN-γ [78]. For the development of CM CAR-T therapy, it has been demonstrated that both CD4^+^ and CD8^+^ T cells contribute to protection [79]. Thus, therapeutic CM CAR-T cells could be engineered to react with coccidioidal parasitic cells by targeting reported spherule surface immunoreactive proteins, such as copper-zinc superoxide dismutase, aspartyl protease, alpha-1,2-mannosidase precursor, and spherule outer wall glycoprotein (SOWgp) or cell wall sugar components [80,81]. Given that CAR-T cells are FDA-approved and have established protocols for clinical-grade production, their application to CM represents a realistic and compelling next step.

Other CAR-based therapeutics include CAR-NK cells, which do not require HLA compatibility, exhibit low safety concerns, and are adaptable to an off-the-shelf therapy setting. Natural killer cells have recently gained interest in invasive fungal infections for their antifungal activity via antibody-dependent cell cytotoxicity (ADCC) by releasing perforin and granzymes, inducing target cell apoptosis, and secreting pro-inflammatory cytokines (e.g., IFN-γ, TNF-α) [82]. Promising prospects for CAR-NK therapy against invasive fungal infections such as candidiasis and cryptococcosis have been demonstrated [83,84]. The scFvκ3-1-CAR-NK-92 cell was engineered to recognize polysaccharides of mannoproteins on the yeast and hyphal surface of *C. albicans* [83,85]. These CAR-NK cells released cytotoxic granules and IFN-γ when incubated with yeast or hyphae of *C. albicans.* Infusion treatment of *C. albicans*-infected NOD scid gamma (NSG) mice with scFvκ3-1-CAR-NK-92 cells decreased the fungal burden in the kidneys [83]. CAR-NK cells developed by Velasco-de-Andres et al. express a lymphocyte scavenger receptor CD5, which can bind fungal cell wall component β-glucans [84]. These NK cells were coined SRCD5CAR-NK, in reference to the second generation of CD5 CAR-NK cells that were designed to potentially combat multiple systemic fungal infections [84]. Infusion of SRCD5CAR-NK into mice infected with either *C. neoformans* or *C. albicans* resulted in decreased fungal burden [84]. Development of anti-CM CAR-NK therapy is plausible since *C. immitis* endospores and young spherules can be inhibited with NK-enriched cells from human PBMC [86].

### 3.3. Checkpoint Inhibitors

Fungal clearance largely depends on the phagocytic ability of innate immune cells [87]. Reports of human, non-human primate, and murine CM have described the infiltration and localization of granulocytes to the proximity of endospores and spherules within the lungs [88,89,90]. Depletion of Ly6G^+^ granulocytes in mice vaccinated with ∆T, caused a significant increase in fungal burden demonstrating neutrophils as key players in *Coccidioides* clearance [49]. In contrast, Ly6G depletion in non-vaccinated mice does not impact fungal clearance, suggesting that the role of Ly6G cells could be multifaceted. Several studies have demonstrated that Ly6G^+^ cells can express the checkpoint inhibitor programmed-death ligand 1 (PD-L1) after exposure to *Coccidioides* [5,75,91].

Immune checkpoint inhibitors, including anti-PD-1, anti-PD-L1, and anti-CTLA-4 antibodies, may control fungal infections by reversing pathogen-induced immune exhaustion and restoring effective host defenses. In severe or chronic fungal infections, persistent antigen exposure often leads T cells to upregulate inhibitory surface receptors, such as PD-1 and CTLA-4. While this upregulation naturally dampens the immune response, preventing excessive inflammation and tissue damage, it can also lead to a hypofunctional state that allows the fungus to growth [92]. Checkpoint inhibitors block these inhibitory signaling pathways allowing exhausted CD4^+^ and CD8^+^ T cells to regain effector functions, proliferate, and secrete protective cytokines, especially IFN-γ [93]. This renewed cytokine production, along with the direct blockade of immune checkpoints on innate immune cells, significantly boosts the phagocytic, oxidative, and fungicidal activities of macrophages and neutrophils [92]. Consequently, check point inhibitor therapy shifts the host immune microenvironment from immunosuppressive tolerance to active defense, improving fungal clearance and significantly increasing survival [93]. Therapy using anti-PD-1, anti-PD-L1, or anti-CTLA-4, has reduced disease severity in mouse models of various pulmonary fungal infections including mucormycosis, aspergillosis, and paracoccidioidomycosis [94,95,96]. A recent study demonstrated that treatment with anti-PD-L1 resulted in moderate but significant survival in *Coccidioides* infected mice. While the exact mechanism contributing to increased survival is still under investigation, these data suggest that it is plausible to develop checkpoint inhibitor therapy against fungal mycoses [91,92].

## 4. Animal and In Vitro Models

The design of preventive and therapeutic immune interventions against CM faces numerous challenges, such as safety, social constructs, turnaround time, financial burden, and model limitations. Different methodologies can complement the development of immune interventions to be more financially advantageous, ensure protective efficacy, translatability, and safety. Here we will highlight animal models, and human organoid work to aid CM vaccine development and immunotherapy.

### 4.1. Humanized Mouse Models

Traditionally, mouse models are the first choice for vaccine development due to the low cost of animals and the ability to use large sample sizes for increased statistical power. However, due to differences in the Major Histocompatibility Complex, epitopes discovered in mice may not always translate into recognizable human epitopes. Therefore, transgenic mice expressing human leukocyte antigen (HLA) closely mimic human antigen recognition, making them valuable for the development of CD4^+^ T cell-based vaccines [97]. For example, transgenic HLA-DR4 (DR4tg) mice containing the allele DRB1*0401 have been used to assess the protective efficacy of live attenuated ∆T against potentially lethal coccidioidal challenge [98]. More importantly, DR4tg mice were used to evaluate the immunogenicity of bioinformatics-predicted human epitopes in the rCpa1 subunit vaccine construct and the vaccine’s protective efficacy for clinical relevance [29,35]. Furthermore, the utilization of humanized mice can uncover potential T cell epitopes that can be validated in human CM patient PBMCs. These epitopes can be further developed for CM diagnosis, vaccination, or CAR-cell targeting.

### 4.2. Non-Human Primates Models

There have been 22 reported cases of naturally acquired coccidioidomycosis in non-human primates (NHPs) housed in endemic regions [88]. Histopathology studies of these infected NHPs revealed a similar influx of cells as seen in humans such as neutrophils, eosinophils, lymphocytes, and plasma cells; however, endosporulation was rarely noted [88]. One limitation of NHP CM models is that NHPs have a different organization of MHC genes than humans, causing differences in HLA class I and II genes. Therefore, NHPs may not accurately represent the landscape of human CD4^+^ T cell clones generated upon vaccination [99,100]. Thus, testing the efficacy of attenuated vaccines may be a more viable option in NHPs than subunit vaccines due to the heterogeneous frequency of T cell clones generated. Despite these drawbacks, NHP models could still be beneficial in testing vaccine candidates, developing immunotherapies, and understanding fundamental mucosal immunology that could bridge the gap between animal and clinical research.

### 4.3. Human Organoids

Human-derived organoids have been developed to complement vaccine development and serve as pathophysiology models for studying infectious diseases. Human peripheral blood mononuclear cells (PBMCs) from patients were widely used for the validation and identification of human T cell epitopes [40]. Human organoids could be a rational next step to aid in research for understanding coccidioidal pathogenesis and developing CM treatment [101,102]. The collection of human PBMCs is less invasive than bronchoalveolar lavage and provides key insights into populations of circulating cells, but whether PBMCs encompass all cells residing in the lungs is uncertain. Lung organoids, consisting of 3D tissue-specific epithelial cells and fibroblasts, were developed to study granuloma formation and macrophage activation for Mycobacterium tuberculosis research by the addition of innate immune cells [103,104]. Wagar et al. further developed an organoid model from healthy adult tonsils, equipped with adaptive immunity for influenza A and B virus (IABV) [101,102,105]. The model was later used to compare immune responses against inactivated IABV vaccine (IIV), live-attenuated IABV vaccine, and a wild-type virus (A/California 2009 H1N1), pioneering vaccine screening in human organoids [101,102]. Similar human organoid systems could be used in CM research including the screening of candidate antigens against different human HLA haplotypes before transitioning into clinical trials and the assessment of immunotherapeutic activity.

## 5. Conclusions

Given that *Coccidioides* has been designated a World Health Organization (WHO) fungal priority pathogen, developing novel therapeutics is urgently needed to mitigate the severity of infections within endemic regions and at-risk populations. With climate change predicted to increase the incidence of CM due to shifting temperature and precipitation patterns, the development of novel vaccines and therapies is particularly critical to combat this foreseeable public health threat [64,106]. The clinical translation of novel immune-based therapies and vaccine candidates holds transformative potential for CM management. Integrating adjunctive immunotherapies could fundamentally shift patient care by moving beyond reliance on prolonged, often toxic, antifungal regimens. This approach may accelerate fungal clearance, reduce the risk of azole resistance, and prevent severe disseminated disease, especially in vulnerable or immunocompromised individuals. Furthermore, a successful prophylactic vaccine would represent a paradigm shift for public health in endemic regions by providing targeted, long-term protection for high-risk populations and occupational workers. As advanced preclinical platforms, such as human immune organoids, improve our understanding of host-specific responses, these immunological insights will pave the way for personalized, host-directed interventions, significantly reducing the clinical morbidity and economic burden of Valley Fever.

## Data Availability

No new data were created or analyzed in this study. Data sharing is not applicable to this article.
